# Genome-Wide Detection of Leukemia Biomarkers from lincRNA–Protein-Coding Gene Interaction Networks in the Three-Dimensional Chromatin Structure

**DOI:** 10.3390/cimb47060384

**Published:** 2025-05-22

**Authors:** Yue Hou, Wei Ning, Muren Huhe, Chuanjun Shu

**Affiliations:** 1 Military Medical Innovation Center, Fourth Military Medical University, Xi’an 710032, China; 2 Department of Bioinformatics, School of Biomedical Engineering and Informatics, Nanjing Medical University, Nanjing 211166, China

**Keywords:** leukemia biomarkers, lincRNA, interaction network, LMAL, GRM7

## Abstract

The human genome is widely transcribed, with part of these transcribed regions producing stably expressed protein-coding or non-coding RNAs. Long intergenic non-coding RNAs (lincRNAs) are significantly differentially expressed in various cell lines and tissues. However, the influence of their transcription events remains unclear. In this study, we constructed a human genomic interaction network and found frequent interactions between lincRNA genes and protein-coding genes that are highly related to the occupancy of RNA polymerase II on the lincRNA gene. Interestingly, in the human genome interaction networks, the degree of lincRNA genes was significantly higher than that of protein-coding genes. The promoter regions of the protein-coding genes interacting with the lincRNA genes are enriched with R-loop structures, indicating that lincRNA may influence the target genes through R-loop structures. These promoters were enriched in more transcription factor binding sites. Furthermore, the whole network and sub-network could be utilized to explore potential biomarkers of leukemia. We found that zinc finger protein 668 (ZNF668), eosinophil granule ontogeny transcript (EGOT), and glutamate metabotropic receptor 7 (GRM7) could serve as novel biomarkers for acute myeloid leukemia (LMAL). Pasireotide acetate (CAS No. 396091-76-2) represents a potential drug for LMAL patients. These results suggested that potential biomarkers and corresponding drugs for cancer could be identified based on lincRNA–promoter network/sub-network topological parameters.

## 1. Introduction

Genes specifying long non-coding RNAs (lncRNAs) occupy a large fraction of the human genome [1]. Many lncRNAs originate from introns of protein-coding genes, overlapping coding sequences, or even as extensions of the 3′ or 5′ ends of genes, while others are transcribed from intergenic regions (lincRNA). Most lincRNA products are non-functional, and they are regarded as transcriptional by-products [2]. Currently, the functions of well-studied lincRNAs can be broadly categorized into four types: serving as markers for specific states or responses, competing with other genomic regions for proteins or microRNAs, assisting target genes in recruiting chromatin-modifying enzymes, or acting as RNA molecular scaffolds for certain proteins [2].

However, the transcriptional expression levels of most lincRNAs are quite low, which is closely related to their high degradation efficiency [3,4]. This indicates that relying solely on lincRNA products to explain their functions is insufficient. Due to the development of chromatin conformation capture techniques, the spatial structure of the genome and its functions have been explored and investigated in depth [5,6]. Transcription plays a crucial role in the chromatin loop extrusion model, where the movement of polymerases can influence the dynamics of chromatin architecture [7]. During active transcription, RNA polymerase II travels along the DNA, creating forces that can facilitate the extrusion of chromatin loops [8]. This process allows for the spatial proximity of distant regulatory elements, such as enhancers and promoters, enhancing the efficiency of gene activation [9]. The transcription machinery can also recruit additional factors that stabilize these loops, promoting long-range interactions within the genome [10,11]. Conversely, when transcription is halted, the forces driving loop extrusion diminish, potentially leading to the reformation of compact chromatin structures [8]. This dynamic interplay underscores the importance of transcription in shaping chromatin topology and highlights its role in regulating gene expression through the modulation of chromatin architecture. However, the function of lincRNA transcription remains unclear.

In this study, we constructed a lincRNA–protein-coding gene interaction network using Hi-C data from the leukemia cell line K562 to systematically analyze the interactions between lincRNA genes and proteins. By integrating GRO-seq, DRIP-seq, and RNA-seq data, we examined the impact of lincRNA transcription on protein-coding genes, thereby identifying key lincRNAs and protein-coding genes associated with leukemia. These results indicated that lincRNA-promoter network/sub-network could be utilized to partially solve the situation regarding that the lack of effective biomarkers and drug targets in tumors.

## 2. Materials and Methods

### 2.1. Protein-Coding Genes and lincRNA Genes

The genomic annotation of protein-coding genes and lincRNA genes in the human genome was downloaded from the GENCODE database [12]. In accordance with previous research [13], promoters were defined as 2 kilobase pairs (kbp) upstream and 0.5 kbp downstream of transcription start sites (TSSs) annotated in GENCODE [12].

### 2.2. Construction of Interaction Network of Protein Genes and lincRNA Genes

Hi-C interaction matrixes of the K562 cell line generated by Rao et al. were downloaded from the GEO repository under accession number GSE63525 [14]. Using the chromatin interactions from Hi-C data, all protein–lincRNA interaction frequencies in the K562 cell line were calculated. To standardize the Hi-C matrix, this study applied the matrix balancing algorithm proposed by Knight and Ruiz to normalize the Hi-C interaction matrix [14]. When the original matrix is not overly sparse, this algorithm, based on an iterative in-out strategy, can effectively balance the matrix. Due to the strong distance bias in Hi-C sequencing, where closer interactions are more likely to be captured, we employed a method to remove the distance effect [14]. First, the possible interaction types (*n*) and the total number of interaction pairs (*N*) at a specific distance (*d*) are counted. Then, the expected interaction intensity at that distance (*E_d_*) is calculated as *E_d_* = *N*/*n*. Finally, each element (*m*_*i*,*j*_) in the Hi-C interaction matrix is divided by the expected value corresponding to the distance *i*-*j*. For a lincRNA gene (*X*) and a protein-coding gene promoter (*Y*), their interaction strength can be calculated using the following formula:

(1) $$M_{X,Y}=\sum m_{i,j}(i\in X;\;j\in Y)$$

where $M_{X,Y}$ is the interaction strength between *X* and *Y*, and $m_{i,j}$ represents normalized Hi-C interaction read counts, with one end located in the promoter region (*Y*) and the other end located in the lincRNA gene body region (*X*).

To calculate statistical confidence estimates for the interaction pairs, *fit-HiC*2 was used to filter Hi-C interactions [15]. Interaction pairs with a false discovery rate (FDR) > 0.001 were excluded.

### 2.3. Network Enhancement

We used the Network Enhancement (NE) tool to denoise our lincRNA–protein interaction networks [16]. NE was applied to enhance the lincRNA–protein interaction networks. The interaction number was input as the network weight. Then, the network was iteratively updated using the NE diffusion process [16].

### 2.4. POLII ChIP-Seq Analysis

The RNA Pol II data of K562 were downloaded from the GEO database (accession number GSE13008). Fastq reads were aligned to hg19 using Bowtie 2.5.2. Peak calling was performed using MACS 2.2.9. A 5 bp window was used around TSSs, and all reads located in the windows were calculated.

### 2.5. Biology Experiments

In wound-healing assay, cells were inoculated into 6-well plates and treated with si-/nc-ZNF668 (siRNA: GUGCCAGCGACUUGCGCAAUU). A straight scratch was made on the plate with a sterilized needle tip when the cell density was approximately 70%. The cell wound edge was marked and photographed under a microscope at the starting time point. After 0 and 24 h, the distance cells migrated was measured and analyzed to determine the wound closure percentage.

For the Transwell assay, cells were inoculated into a 24-well Transwell cell apical chamber containing matrix gel (BD, Franklin Lakes, NJ, USA) to evaluate migration. The bottom and upper chambers contained the RPMI medium and serum-free medium, respectively. Cells that invaded the bottom chambers were fixed with 4% polyformaldehyde, stained with 0.1% crystal violet solution, and then photographed under Olympus XC50 camera which is produced in Tokyo of Japan.

### 2.6. DRIP-Seq Data Analysis

DRIP-seq data of K562 cell lines were downloaded from the GEO database under accession number GSM7061506. Trimmed fastq reads were aligned to hg19 using Bowtie2. Peak calling was performed using MACS2. To create DRIP-seq coverage plots, the locations of the mapped DRIP-seq reads were extended to 150 bp to represent sequenced fragments, normalized (to RPKM), and reformatted in the bigWig file format.

### 2.7. Gene Enrichment Analysis

We extracted the top 3000 genes with highest degree in this network and performed GO (Gene Ontology) analysis [17]. We utilized the well-adopted hypergeometric test and Benjamini–Hochberg *p*-value correction algorithm to identify all ontology terms. We used 10,000 random selected genes as background genes.

### 2.8. TCGA Data Download, Processing, and Analysis

The mRNA expression data and clinical data of cancer patients were downloaded from TCGA database (https://genome-cancer.ucsc.edu/, accessed on 17 May 2025). These expression data were firstly normalized, and differential expression analysis was then performed for SEMA3F using the R package limma 3.20 [18]. A *p*-value < 0.05 was considered as statistically significant. According to clinical data, we estimated cumulative survival curves and overall survival rates using Kaplan–Meier curves. Then, hazard ratios (HRs) and corresponding 95% CIs were estimated based on Cox proportional hazard models. Indeed, higher HR values (HR > 1.0) indicate bad prognosis, while lower HR values (HR < 1.0) indicate good prognosis. *p* values were calculated using the Log rank test.

### 2.9. Single-Cell RNA Analysis

The single-cell RNA sequencing data were downloaded from the NCBI GEO database for leukemia [19]. Six single-cell RNA sequencing datasets, i.e., GSE142213, GSE132509, GSE116256, GSE147989, GSE111104, and GSE125881, were used in this study. These six scRNA-seq datasets were firstly normalized using the harmony algorithm in Seurat. The scRNA-seq data analysis process was consistent with that used in our previous study [20]. Cell phenotype and macrophage polarization experiments were repeated three times and analyzed using unpaired *t*-tests. Differences were considered statistically significant when *p* < 0.05 or log-rank *p* < 0.05.

### 2.10. The Interaction Between lincRNA and miRNA

MicroRNAs (miRNA) bind to lincRNAs and mRNAs to regulate the expression of RNAs. To uncover lincRNAs involved in this mechanism, we studied the interactions between miRNA and lincRNA. To do this, we ran mi-Randa and detected the miRNA binding sites in reference lincRNA sequences. Furthermore, the interactions between miRNA and lincRNA were also predicted using TargetScan 8.0 and RNAhybrid 2.1.2. Then, the overlapping prediction interactions between lincRNAs and miRNAs were identified as reliable interactions. According to the free energy in mi-Randa, the top 10 miRNAs for lincRNA were chosen.

### 2.11. Potential Small Molecule Drugs Targeting GPCR

The full atoms for the GPCR (G protein-coupled receptor) were predicted using Alpha Fold 3 [21]. The small molecules were obtained from FDA-approved drugs. Then, 1972 small molecules were obtained. The corresponding 3D structures were downloaded from the PubChem database. Then, the largest possible binding pocket of GRM7 was predicted using Discovery Studio 3.0. These predicted pockets were utilized to construct an initial coarse model of the protein–molecule complex. Then, interaction between small molecules and protein were by further explored using Discovery Studio 3.0. Based on binding energy scores, the complex with the lowest score was chosen as the finally structure. High-quality 3D images of structures were drawn using Discovery Studio 3.0 and PyMOL 3.0.

## 3. Results

### 3.1. LincRNA–Protein-Coding Gene Interaction Network

**Figure 1:**
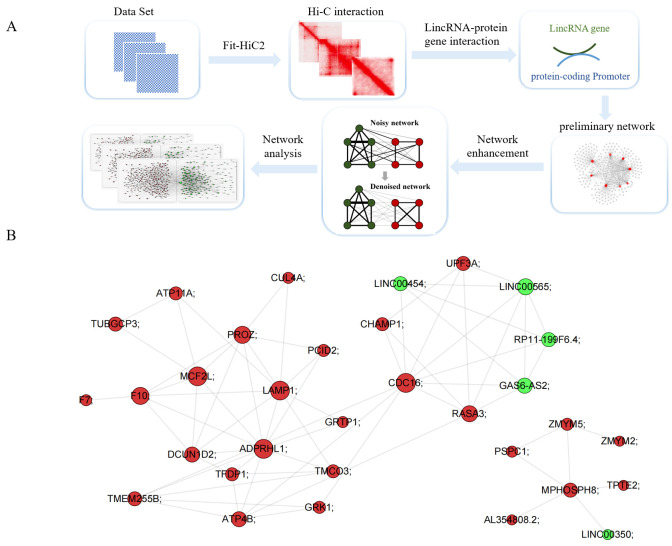
**Construction of the lincRNA–protein-coding gene interaction network.** (**A**) The workflow for constructing the lincRNA–protein-coding gene interaction network. (**B**) Part of the lincRNA–protein-coding gene interaction network for chromosome 13. The green nodes indicate lincRNA genes. The red nodes indicate the promoters of protein-coding genes. The size of each node represents the degree of this node in the interaction network. The grey lines between the nodes indicate the interaction frequency.

Hi-C was used to construct a human genome interaction network that includes all protein-coding gene promoters as well as lincRNA genes. We used the number of Hi-C interaction sequencing fragments to assess the strength of the interaction between the two elements, i.e., lincRNA and promoter. In order to remove false-positive interaction pairs, *fit-HiC*2 was utilized to calculate the FDR (false discovery rate) of all interaction pairs (Figure 1A). Then, the network was enhanced using Network Enhancement (NE) tools [16]. NE, a method for improving the signal-to-noise ratio of undirected, weighted networks, was adopted to remove weak edges and enhance real connections. NE tools alleviates interpretation of noisy Hi-C contact maps from the human genome. The workflow for constructing the lincRNA–protein-coding gene interaction network is shown in Figure 1A. Using all protein-coding genes and lincRNA genes annotated by the GENCODE database [12,22], we calculated the human genome interactions between lincRNA genes and protein-coding genes.

For leukemia, the Hi-C data were downloaded from the GEO (Gene Expression Omnibus) database (GSE63525). Then, the lincRNA–promoter network was constructed based on the workflow in this study (Figure 1A,B). This network consisted of 25,006 nodes. A node and edge in the lincRNA–protein-coding gene interaction network represents the gene and spatial interaction between genes. Among these nodes, there are 214,340 interaction relationships (Supplementary Materials Excel S1). The diameter of the network is 22. On average, each node interacts with 17 nodes. Figure 1B presents a sample of the lincRNA–protein-coding gene interaction network, i.e., a part of the network of chromosome 13. Network interaction analysis revealed distinct topological preferences among gene categories: interactions between lincRNA genes accounted for 36.7% of all observed connections, while protein-coding gene interactions comprised 42.7% of the total network. The remaining 20.6% of interactions represented cross-regulatory associations between lincRNA and protein-coding genes, demonstrating their functional interconnectivity. The count of interaction pairs among different chromosomes is shown in Supplementary Materials Figure S1A.

### 3.2. Topology Analysis of the lincRNA–Protein-Coding Gene Interaction Network

**Figure 2:**
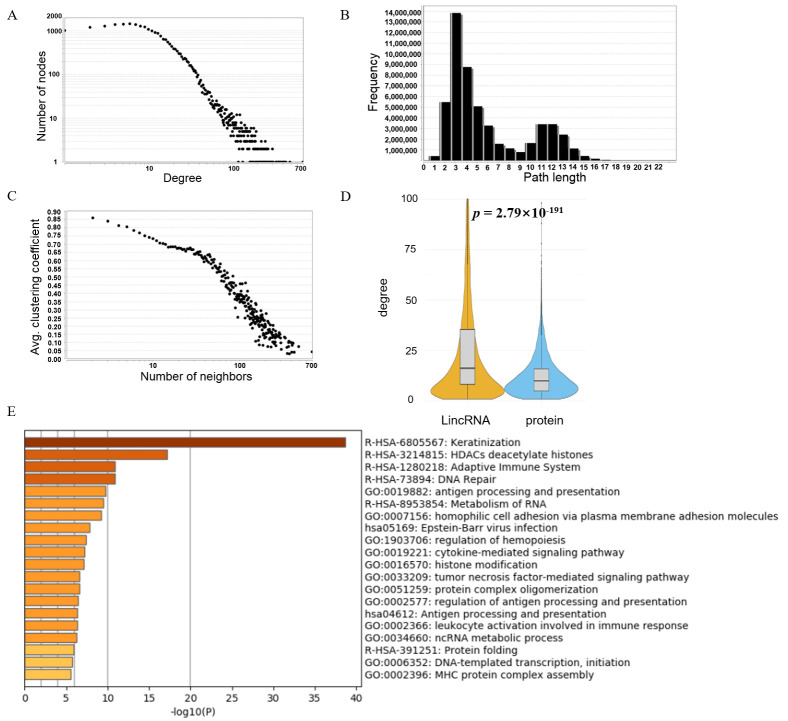
**The topological parameters for the lincRNA–promoter network in leukemia.** (**A**) The number of the genes that are in different degrees. (**B**) The number of paths that are in different length. (**C**) The average clustering coefficient in the human genome interaction network. (**D**) Boxplot of the degrees of lincRNAs and protein-coding genes. (**E**) GO enrichment analysis for lincRNA-related proteins.

To understand the biological significance of the network, the whole interaction network topological parameters, i.e., degree, path length, number of neighbors, clustering, closeness centrality, betweenness centrality, and expression level, were analyzed. As shown in Figure 2A, the number of genes gradually decreases with increasing degree, suggesting that most genes exhibit limited interaction ranges. Only a small subset of core genes engage in extensive interactions. These core genes, with their broad interaction networks, are the primary focus of our study. The number of genes in the network with a degree ≤ 10 was the highest, which is similar to that reported in a previous study [23]. Network topology analysis revealed a maximum geodesic distance of 22 edges between diametrically opposed nodes, indicating extended signaling potential. Conversely, paths spanning three edges constituted the most abundant interaction motif (Figure 2B), highlighting preferential short-range connectivity patterns.

By comparison, we found that the average degree for a lincRNA gene was 31.82, while that of a protein-coding gene was 12.17 (Figure 2D, the Student’s *t*-test *p* = 2.79 × 10^−191^) suggesting that lincRNA genes were more likely to interact with other genes in three-dimensional space (Supplementary Materials Figure S1B–D). Interestingly, a previous study used microRNA genes and protein-coding genes to build a human genome interaction network using ChIA-pet interaction data, and the degree of microRNA genes in the network was also significantly higher than that of protein-coding genes [24]. These results suggested that ncRNA genes frequently make contact with other genes.

We extracted the top 3000 genes with the highest degrees in this network and performed GO (Gene Ontology) analysis [17]. The enriched results showed that these most interacting genes were involved in various important biological processes, including regulation of histone modification, immune response, DNA repair, RNA metabolism, ncRNA metabolic processes, protein folding, and DNA transcriptional initiation (Figure 2E). Histone deacetylase (HDAC) pathways mediate histone deacetylation, silencing tumor antigen genes such as MHC-I to promote immune evasion, while lincRNAs XIST and NEAT1 dynamically modulate immune checkpoint molecules or B-cell antigens by binding HDAC1 complexes or suppressing HDAC6 activity, thereby influencing CAR-T efficacy. The adaptive immune dysfunction pathway is further exacerbated by lincRNA HOTAIR, which recruits HDACs to suppress antigen-processing genes, and PVT1, which stabilizes PD-L1 mRNA to enhance T-cell resistance. Genomic instability arises from defects in DNA repair pathways. Additionally, aberrant activation of the keratinization pathway, driven by KRT7-AS-mediated overexpression of KRT7, facilitates cytoskeletal remodeling and bone marrow infiltration in acute myeloid leukemia. These findings collectively underscore lncRNA-mediated crosstalk among epigenetic silencing, immune checkpoint dysregulation, and DNA repair defects, providing a rationale for targeting lincRNA-HDAC interactions in therapeutic strategies.

### 3.3. Effects of lincRNA on Chromatin Status Around Target Genes

**Figure 3:**
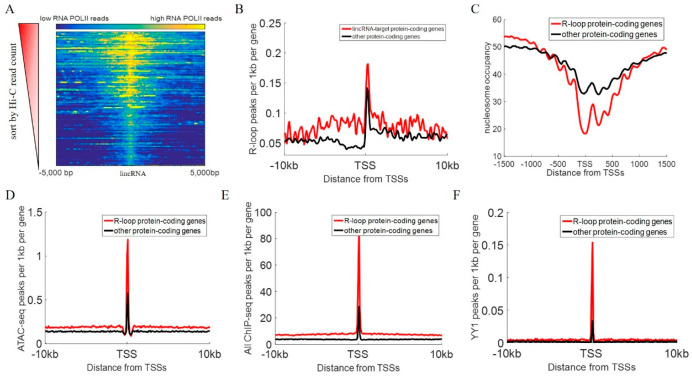
**The epigenetic features around lincRNA-targeted protein-coding genes.** (**A**) Heatmaps of RNAPII ChIP-seq read counts around lincRNA genes. Each row represents RNAPII ChIP-seq read counts around one lincRNA gene. All lincRNA genes were sorted by their total Hi-C read counts. (**B**) The distribution of R-loop peaks around protein-coding genes. (**C**) Nucleosome occupancy around protein-coding genes. (**D**) The distribution of ATAC-seq peaks around protein-coding genes. (**E**) The distribution of all TF ChIP-seq peaks around protein-coding genes. (**F**) The count of YY1 ChIP-seq peaks around protein-coding genes.

To explore the potential mechanism for interaction between lincRNAs and promoters, the chromatin structure around target genes interacting with lincRNAs was analyzed. We found that the interaction strength of lincRNA genes was positively correlated with the RNA POLII occupancy of lincRNA genes (Figure 3A, Spearman correlation R = 0.84, *p* = 4.52 × 10^−84^), indicating that lincRNA interaction strength was highly correlated with RNA POLII enrichment. A previous study has shown that the 5′ region of active lincRNA genes is enriched with a large number of DNase 1 hypersensitivity sensitive sites, H3K4me3 histone modifications, and RNA POLII occupation [25], which are very similar to the transcriptional characteristics of protein-coding genes. In addition to the function of transcriptional genes, RNA POLII is also one of the important proteins mediating chromatin interactions [26]. This result indicated that RNA POLII, which is required for lincRNA transcription, is one of the key factors mediating its chromatin interactions.

The R-loop structure is a nucleic acid structure formed by the hybridization of RNA and single-stranded DNA, which releases another single-stranded DNA [27]. It is very common in the human genome, and about 5% of genomic regions have R-loop structures [28]. LncRNAs can form R-loop structures with target genes using the mode *in* *trans*, thereby exercising its regulatory function [29]. As shown in Figure 3B, the promoter regions of protein-coding genes that interact with lincRNA genes are enriched with R-loop signals (average DRIP-seq TPM signal value = 0.183), while the promoter regions of other protein-coding genes have significantly lower R-loop signals (average DRIP-seq TPM signal value = 0.146, Student’s *t*-test, *p* = 2.98 × 10^−36^). However, there was no significant difference in the number of R-loops contained in the two proteins-coding genes in the downstream genomic regions of TSSs (Student’s *t*-test, *p* > 0.01). Therefore, we inferred that the spatial contact between lincRNA genes and target genes makes it easier for lincRNA to bind to the target gene promoter region through an R-loop mechanism.

Furthermore, all protein-coding genes interacting with lincRNAs were identified using the human genome interaction network combined with R-loop high-throughput sequencing data (DRIPc-seq [27]). When target gene promoter regions contain DRIPc-seq peak regions, we identify these protein-coding genes as the target genes regulated by lincRNA via R-loops, hereinafter referred to as R-loop protein-coding genes. To compare the chromatin status of R-loop protein-coding genes and other protein-coding genes, we calculated the distribution of nucleosomes in their promoter regions and the data of chromatin open regions, respectively (Figure 3C,D). As shown in Figure 3C, the transcription initiation sites of R-loop protein-coding genes have a significantly low nucleosome-free region (Student’s *t*-test, *p* = 9.33 × 10^−31^). The +1 nucleosome downstream of the R-loop protein-coding gene TSS is also better located. Nucleosome-free regions near TSSs ensure that DNA remains open and can bind to various proteins, including various chromatin regulators, transcription factors, and transcription machinery [30]. The +1 nucleosome is important for gene transcription because it recruits transcription factors to regulate the occurrence of transcriptional events [31,32,33]. Therefore, the promoter region of the R-loop protein-coding gene is in a more open environment and facilitates the recruitment and assembly of transcription machinery. In addition, the ATAC-seq data also indicated that the R-loop protein-coding gene promoter region was enriched with more ATAC-seq peak regions (Figure 3D). These results suggested that lincRNA-related R-loops were potentially correlated with the activation of target protein-coding genes.

Due to the open chromatin environment of the R-loop protein-coding gene promoter region (Figure 3C,D), transcription factors can be relatively easily bound to this region. The R-loop protein-coding gene promoter region is significantly enriched with transcription factor binding sites (TFBSs, Figure 3E, Student’s *t*-test, *p* = 8.46 × 10^−118^). We used the ReMap online analysis tool to integrate 301 datasets for the transcription factor ChIP-seq dataset of the K562 cell line in the ENCODE project [34]. Then, we found that the number of TFBSs (average count = 84.67) in the promoter regions of R-loop protein-coding genes was significantly higher than that of other protein-coding genes (average count = 27.22, Student’s *t*-test, *p* = 8.46 × 10^−118^). YY1 is a transcription factor that can be recruited by transcriptional products [35]. The enrichment of a large number of YY1 transcription factor binding sites in the promoter region of R-loop protein-coding genes indicated that lincRNA acting on the promoter region of target genes in the form of an R-loop will help target genes recruit a large number of YY1 transcription factors (Figure 3F). These results suggested that lincRNA functions on target gene promoters through R-loops; thus, the promoter region was enriched with a large amount of TFBSs.

Although our findings suggest that R-loops may play a role in the regulation of target genes based on DRIP-seq data, it is important to note that the current study does not provide experimental evidence to establish whether these interactions are causative or merely correlative. Future experimental perturbations, such as R-loop inhibition or lincRNA knockdowns, would be necessary to clarify the nature of these interactions.

### 3.4. Biological Significance of the lincRNA–Promoter Network in Leukemia

**Figure 4:**
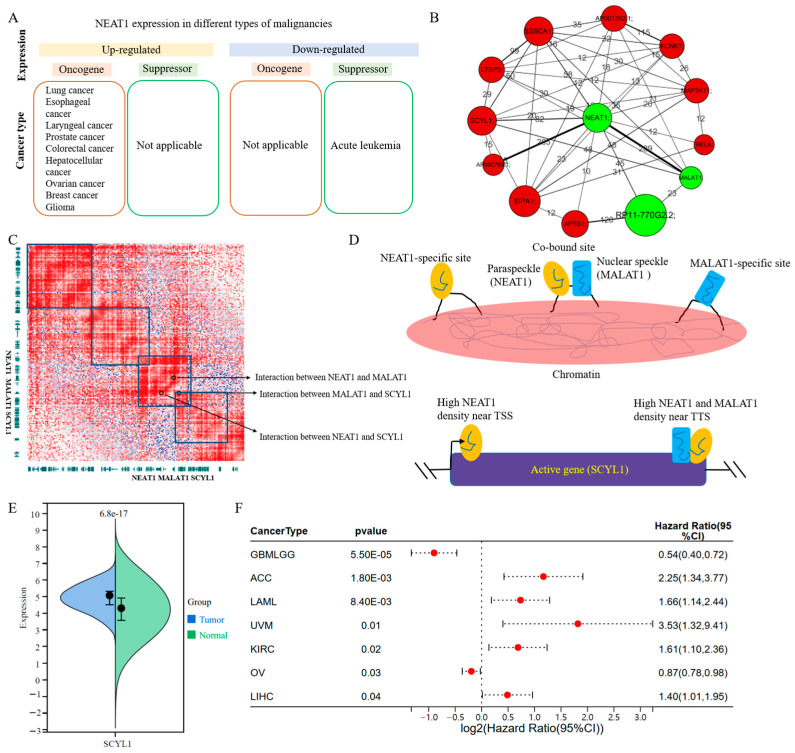
**Part of the interaction network of chromosome 11 in the K562 cell line.** (**A**) NEAT1 pan-cancer function. (**B**) The sub-network for NEAT1 in LAML. Green dots and red dots represent lincRNA genes and protein-coding genes, respectively. The size of the dots represents degree in this network. The values and the sizes of lines among dots indicate the interaction frequency. (**C**) The interactions among NEAT1, MALAT1, and SCYL1. (**D**) The model for NEAT1 and MALAT1 functions in protein-coding genes. (**E**) The expression of SCYL1 in LAML tissues and corresponding normal tissues (log2 (TPM+1), tumor: 173; normal: 70). (**F**) The significance HR values of SCYL1 in a variety of cancers.

To further verify biological significance of the lincRNA–promoter network, the sub-network of genes that are known to be associated with leukemia was chosen for analysis. NEAT1 is a lincRNA gene that acts as a tumor suppressor in acute leukemia, and its expression level directly affects the differentiation of white blood cells (Figure 4A) [36,37]. Hence, we selected all genes (lincRNA and promoter) that directly interact with NEAT1 to construct the sub-network (Figure 4B). According to the number of Hi-C interaction counts, a close interaction between NEAT1 and the lincRNA gene MALAT1 was found (the number was 289) (Figure 4B). The MALAT1 gene is involved in the regulation of the differentiation and expansion of leukemia cells [38]. The frequent contact between MALAT1 and NEAT1 in 3D space is potentially related to the functional interaction between these two proteins in leukemia cells (Figure 4C). A study using CHART technology has shown that lincRNA products of MALAT1 and NEAT1 are widely co-bound to the same chromatin region, at least in hundreds of genomic regions (Figure 4D) [39]. These results indicated that MALAT1 and NEAT1 interact closely at the chromatin level, thus providing favorable conditions for their transcripts to accurately co-locate to chromatin targets.

Based on the sub-network, we found that both NEAT1 and MALAT1 genes interact with the protein-coding gene SCYL1 (Figure 4B,C). It has shown that removing the promoter region of MALAT1 will have a significant impact on the expression levels of SCYL1 and NEAT1 genes [40]. Our results further validate the interaction of these genes at the chromatin level in LAML. Moreover, SCYL1 is significance highly expressed in tumor tissues, when compared to normal tissues in LAML (*p* = 6.8 × 10^−17^) (Figure 4E). SCYL1 is an oncogene for LAML based on Cox proportional hazards model analysis results (Figure 4F, Supplementary Materials Figure S2). Furthermore, the function of SCYL1 is still unknown in LAML. Hence, these results suggested that the potential synergistic action of NEAT1 and MALAT1 regulates the expression of SCYL1, which is a novel biomarker for LAML. The sub-network was not only utilized to verify the known biological functions of lincRNA genes in LAML, but also could be utilized to find novel protein biomarker.

### 3.5. Potential Biomarkers of Leukemia from GPCRs Based on the lincRNA–Promoter Network

**Figure 5:**
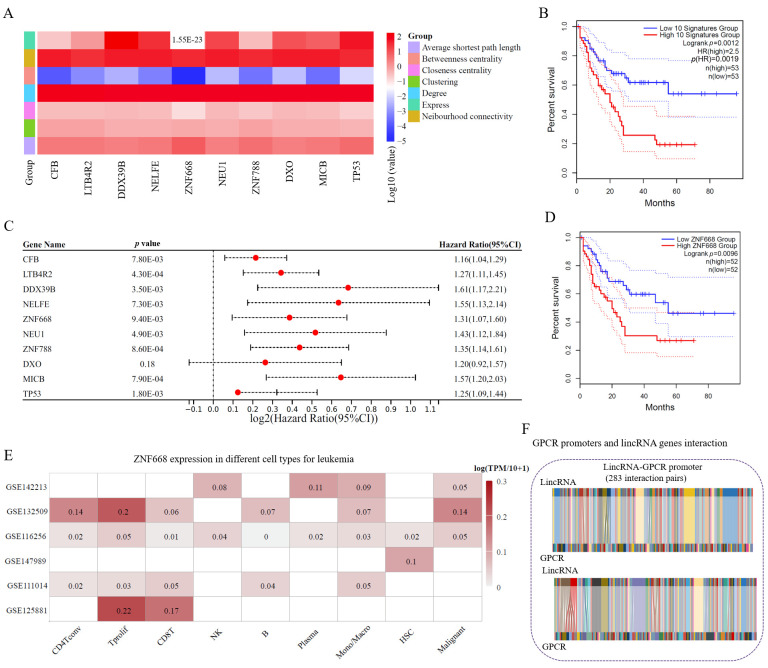
**Exploring potential biomarkers from the network topological parameters analysis.** (**A**) Topological parameters for the proteins with top 10 degrees in the lincRNA–promoter network. The log10 values for each topological parameters were calculated and represented in a heat map. Outliers are displayed directly as values (same below). (**B**) Kaplan–Meier overall survival plots for the 10 signature groups in LAML. (**C**) HR values for the 10 signatures in LAML. (**D**) Kaplan–Meier overall survival plot for ZNF668 in LAML. (**E**) ZNF668 expression in different leukemia cell types. (**F**) Interaction landscape of GPCR promoters and lincRNA genes.

NEAT1–SCYL1 is a lincRNA–promoter interaction pair that could be utilized to determine the prognosis of LAML patients. However, it is challenge to explore biomarkers from the lincRNA–promoter network since it has more than one hundred thousand interaction relationships. Here, we used two approaches to explore potential biomarkers. On the one hand, it could be chosen based on sorted network topological parameters scores. On the other hand, it could be obtained based on the extraction and analysis of sub-networks. The topological parameters for proteins with the top 10 degrees in the lincRNA–promoter interaction network of LAML are listed in Figure 5A (Supplementary Materials Table S1). According to gene expression and clinical parameters, it was found that LAML patients with a low expression for these 10 signature genes always have a better prognosis when compared with LAML patients with high expression (Figure 5B). Meanwhile, 9 of the 10 proteins have a significant HR (hazard ratio) value (Figure 5C). A half of these proteins have no reported relationship with leukemia, i.e., LTB4R2, DDX39B, ZNF668, ZNF788, and DXO. However, HR values and survival analysis results indicated that these proteins probably act as oncogenes in leukemia patients, suggesting that these proteins probably act as novel biomarkers for LMAL.

Furthermore, two proteins among these top 10 proteins belong to zinc-finger proteins (ZNFs). One ZNF (ZNF668) was chosen to analyze it role in the progression of LAML based on data from TCGA and GEO databases. We found that it is an oncogene in LAML patients, and it is highly expressed in T and malignant cells (Figure 5D,E, Supplementary Materials Figure S3). Transwell assays and would healing assays were performed to further verify the oncogene roles for ZNF668 in K562 cells. Based on the results of biological experiments, we found that ZNF668 promotes cell proliferation and migration in K562 cells (Supplementary Materials Figure S4). It is suggested that the high ZNF668 expression could promote cancer cell proliferation and migration for leukemia (Supplementary Materials Figure S4). These results suggested that ZNF668 acts as an oncogene and influences the tumor immune microenvironment in LAML. The roles of ZNFs in the development, progression, and metastasis of malignant tumors via regulating gene transcription and translation processes are evident. Therefore, ZNF668 and ZNF788 should be favored by tumor biologists (Supplementary Materials Figures S3–S5), especially in LAML-related researchers.

**Figure 6:**
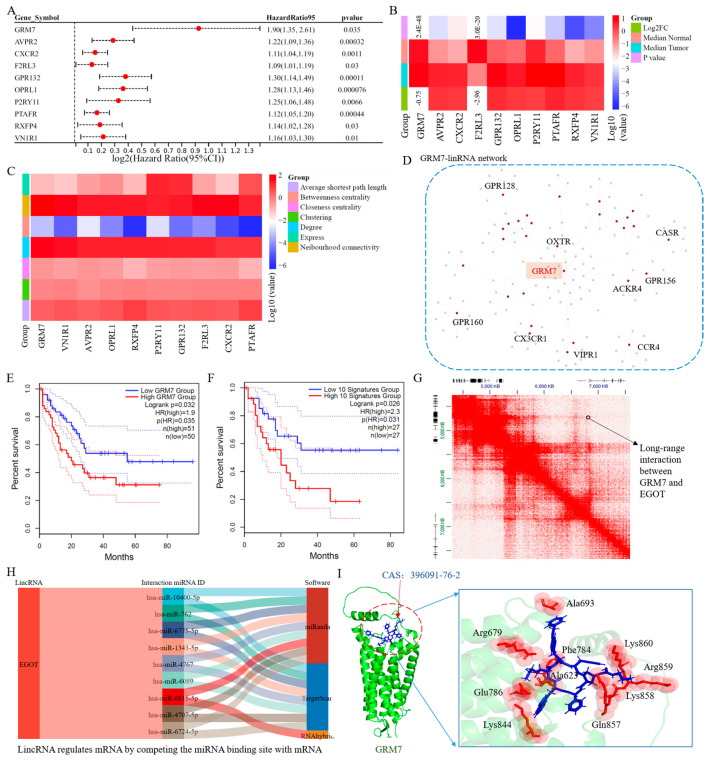
**GPCRs promoter–lincRNA network for LAML.** (**A**) HR values for GPCRs with the top 10 degrees in the lincRNA–promoter interaction network of LAML. (**B**) mRNA expression of the top 10 GPCRs. (**C**) The topological parameters for the top 10 GPCRs. (**D**) GRM7–lincRNA network. (**E**) Kaplan–Meier overall survival plot for ZNF668 in LAML. (**F**) Kaplan–Meier overall survival plot for the top 10 GPCRs in LAML. (**G**) The interaction between GRM7 and EGOT. (**H**) The lincRNA and miRNA interaction pairs. (**I**) Potential molecular drugs for GRM7 identified from FDA-approved drugs.

G protein-coupled receptors (GPCRs) are the largest and most diverse protein family in the human genome and have become the most successful drug target class in pharmaceuticals. Based on expression and clinical parameters for LAML, the HR values and log2FoldChange (log2 (Tumor/Normal expression)) for each GPCR were calculated. Hence, to explore potential targets for LAML, the GPCR–lincRNA networks were extracted based on GPCRs that not only have significant HR values but also exhibit differences in gene expression between tumor and normal tissue (Figure 5F and Supplementary Materials Figure S6). Then, the topological parameters for these GPCRs were also computed. We screened the top ten GPCRs according to the reverse order of degrees. The corresponding HR values, expression, and topological parameters for these GPCRs are shown in Figure 6A–C (Supplementary Materials Tables S2 and S3). GRM7 had the highest degree in the GPCR–lincRNA network, and the corresponding sub-network is shown in Figure 6D.

GRM7 probably acts as a novel biomarker for LAML patients based on the Kaplan–Meier overall survival plot. The Kaplan–Meier overall survival plot for the top 10 GPCRs in LAML show that these GPCRs could be further applied to establish a prognostic risk model (Figure 6F). Furthermore, the main lincRNA that interacts with GRM7 with a high frequency is EGOT (Figure 6G). EGOT is a crucial regulator in the most cancers, such as liver and thyroid cancer [41,42]. Meanwhile, a large number of RNA therapies were in phase II or III clinical experiments, including newer entities such as miRNA mimics. However, no lncRNA-based therapies were reported [43]. Hence, based on binding energy scores, miRNAs that interact with EGOT are shown in Figure 6H. miR-10400-5p is a miRNA; it has the lowest energy when it interacts with EGOT (Figure 6H, Supplementary Materials Table S4). Furthermore, we predicted 3D structures of GRM7 using Alpha Fold 3 [21]. Then, molecular drugs that could potentially inhibit the expression of GRM7 were identified based on computer-based virtual screening of 1792 FDA-approved small molecule drugs. Using Rosetta and Discovery Studio 3.0 software, the best small molecule drug with the highest docking score was pasireotide acetate (CAS No. 396091-76-2) (Figure 6I). The interaction sites between pasireotide acetate and GRM7 re shown in Figure 6I. These results suggested that potential biomarkers and corresponding drugs could be identified based on sub-network analysis of a set of genes with drug-targeting potential.

## 4. Discussion

Molecular cancer biomarkers are any measurable molecular indicator of risk of cancer, occurrence of cancer, or patient outcome [44]. They may include somatic genetic variants, epigenetic signatures, transcriptional changes, and proteomic signatures [45]. These indicators are based on biomolecules, such as nucleic acids and proteins [20,46]. However, the process used for the identification of indicators always ignores the lincRNA–protein-coding gene interaction network in the three-dimensional chromatin structure. In this study, we proposed a method for constructing a lincRNA–protein-coding gene interaction network to explore biomarkers for leukemia.

By constructing and analyzing the human genome interaction network (Figure 1A,B), we found that lincRNA genes frequently interact with other genes under the three-dimensional structure of chromatin, and the number of lincRNA genes in the human genome interaction network is significantly higher than that of protein-coding genes (Figure 2), indicating that lincRNAs frequent interact with other genomic regions. We discovered that the interaction intensity of lincRNA genes with other genes is positively correlated with RNA POLII occupancy levels (Figure 3A). Additionally, the promoter regions of lincRNA target genes are enriched with a substantial number of R-loop structures (Figure 3B). Protein-coding genes regulated by this pattern exhibit notable nucleosome depletion zones and numerous TFBSs surrounding their transcription start sites (TSS, Figure 3C–F).

Although, the causal relationship between R-loops and TFBSs in the occurrence and development of leukemia requires further experimental validation, several studies have proved that abnormal R-loops directly lead to subsequent immune activation. Overexpression of the R-loop resolving enzyme prevented cytosolic ssDNA accumulation and ARID1A interferon gene upregulation [47]. ARID1A loss in tumor cells induces R-loops, which give rise to cytosolic DNA species that activate STING-type I IFN signaling, inducing an ARID1A–IFN gene expression signature that promotes anti-tumor immunity [47]. Moreover, the enrichment of R-loops can predict survival outcomes and treatment responses [48]. R-loop distribution potentially participates in LUAD progression by affecting the Ras signaling pathway [48]. In addition, several neurological disorders, including ataxias, neuromuscular disorders, and nucleotide repeat expansion disorders, result from mutation of genes involved in R-loop resolution [49]. Our results suggested a potential model wherein R-loops influence TFBSs around the hub-genes in the interaction network, leading to the development of leukemia.

To verify the biological significance of the lincRNA–promoter network, we found that two genes closely related to the differentiation of leukemia cells, NEAT1 and MALAT1, interact closely at the chromatin level (Figure 4A–C). Moreover, the transcription products of these two lincRNA genes also act simultaneously on the same genomic regions, indicating that the proximity of the spatial distance of chromatin provides favorable conditions for their transcription products to co-localize at genomic targets [39]. For example, they function on the SCYL1 promoter, and its corresponding protein was identified as a novel biomarker for LAML in this study.

To further explore the biological significance of the lincRNA–promoter network, potential biomarkers for LMAL were identified based on network topological parameter analysis. For the whole network and GPCR sub-network, proteins with top 10 degrees in the lincRNA–promoter interaction network of LAML were further applied to establish a prognostic risk model. The top 10 proteins also act as survival prognostic factors for LAML patients. Meanwhile, some proteins act as known oncogenes in most cancers except LAML, such as ZNF668 and GRM7 [50,51]. A potential small molecule drug targeting GRM7 is pasireotide acetate (CAS No. 396091-76-2), according to the results of our computer-assisted drug design. Of course, the protein-linked lincRNAs were also related to cancer development, such as EGOT [41,42]. These results indicated that the lincRNA–promoter network could act as an effective pathway to explore novel potential biomarkers for cancer.

In acute myeloid leukemia (AML) patients, both genetic and pharmacological inhibition of STING lead to reductions in AE leukemia cells, indicating that STING is crucial for leukemia cells [52]. The formation of the R-loop structure leads to the activation of STING-dependent cytosolic DNA sensing [47]. Furthermore, anti-tumor immunity is dependent on the cGAS-STING cytosolic DNA sensing pathway [47]. R-loops can also interact with DNA to activate innate immune responses, including TLR3 and cGAS-STING. When R-loops are deregulated or resolution pathways are disrupted, some genomic R-loops become susceptible to nucleolytic processing, raising the levels of cytoplasmic hybrids above a critical threshold for IRF3 activation [53]. At the same time, an increase in RNA–DNA hybrids in the cytoplasm also leads to an increase in apoptosis [53], and STING has recently been reported to be associated with apoptosis and inflammation [53,54]. STING deficiency or inhibition of PARP1 function can reduce the expression of the proapoptotic gene PUMA, decrease the localization of Bax on the mitochondrial membrane, and thus reduce cell apoptosis [54].

## Data Availability

The authors confirm that the data supporting the findings of this study are available within the article and its Supplementary Materials. The original data used to support the findings of this study are available from the corresponding author upon a reasonable request by e-mail.

## Supplementary Materials

The supporting information can be downloaded at: https://www.mdpi.com/article/10.3390/cimb47060384/s1.
