# Supplementary material for: Genome-Wide Detection of Leukemia Biomarkers from lincRNA–Protein-Coding Gene Interaction Networks in the Three-Dimensional Chromatin Structure
Source: Curr Issues Mol Biol. 2025 May 22;47(6):384. doi: 10.3390/cimb47060384 (PMC12191658; doi:10.3390/cimb47060384)
Supplement: Supplementary file 1 [file cimb-47-00384-s001.zip › Supplementary materials.pdf]

# Genome-wide detection of leukemia biomarkers from lincRNA–protein-coding gene interaction networks in the three-dimensional chromatin structure

Yue Hou [1,†], Wei Ning [1], Muren Huhe [1] and Chuanjun Shu [2,*,†]

[1]Military Medical Innovation Center, Fourth Military Medical University, Xi'an 710032, China.

[2]Department of Bioinformatics, School of Biomedical Engineering and Informatics, Nanjing Medical University, Nanjing 211166, China.

*Correspondence: chuanjunshu@njmu.edu.cn

† These authors contributed equally to this work.

A

| | lincRNA-protein | lincRNA-lincRNA | protein-protein |
|---|---|---|---|
| **chr1** | 4192 | 3673 | 9304 |
| **chr2** | 2048 | 2574 | 3693 |
| **chr3** | 1622 | 1565 | 3779 |
| **chr4** | 722 | 897 | 1201 |
| **chr5** | 1377 | 2043 | 2620 |
| **chr6** | 1897 | 1500 | 5729 |
| **chr7** | 1331 | 1255 | 2839 |
| **chr8** | 1365 | 1976 | 1825 |
| **chr9** | 1426 | 1139 | 3594 |
| **chr10** | 1117 | 1319 | 1940 |
| **chr11** | 3260 | 2664 | 7943 |
| **chr12** | 2304 | 2239 | 4707 |
| **chr13** | 388 | 521 | 516 |
| **chr14** | 1381 | 1623 | 2731 |
| **chr15** | 1307 | 1680 | 1929 |
| **chr16** | 3568 | 3869 | 5962 |
| **chr17** | 4515 | 4564 | 8752 |
| **chr18** | 461 | 1157 | 387 |
| **chr19** | 6259 | 4180 | 14575 |
| **chr20** | 981 | 846 | 2619 |
| **chr21** | 665 | 957 | 877 |
| **chr22** | 1393 | 1292 | 2353 |
| **chrX** | 437 | 244 | 1733 |

B

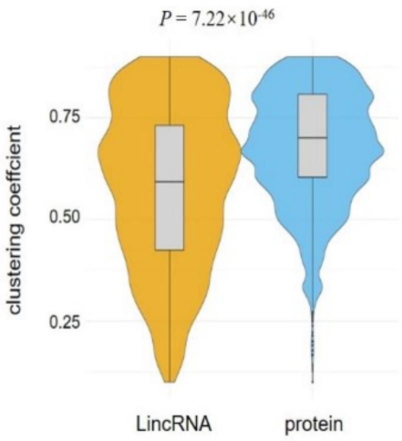


C

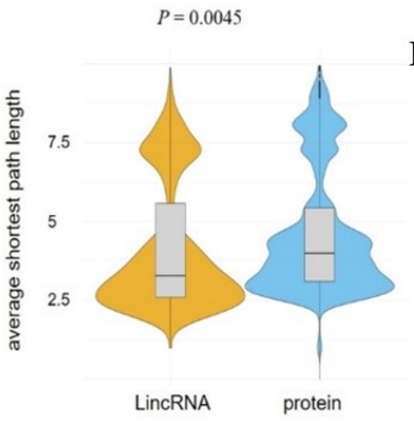


D

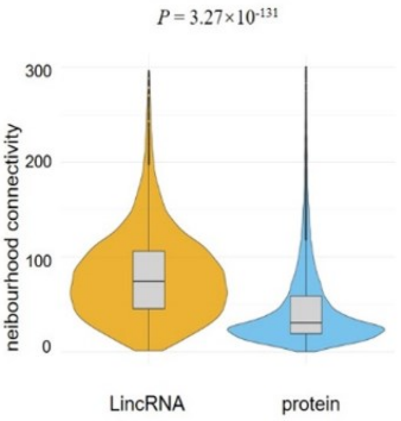


Figure S1. Topological parameters for lincRNA and protein. (A) Count of different types of interactions along chromosomes. (B) Clustering coefficient values for lincRNA and protein. (C) Average shortest path length values for lincRNA and protein. (D) Neighborhood connectivity values for lincRNA and protein.

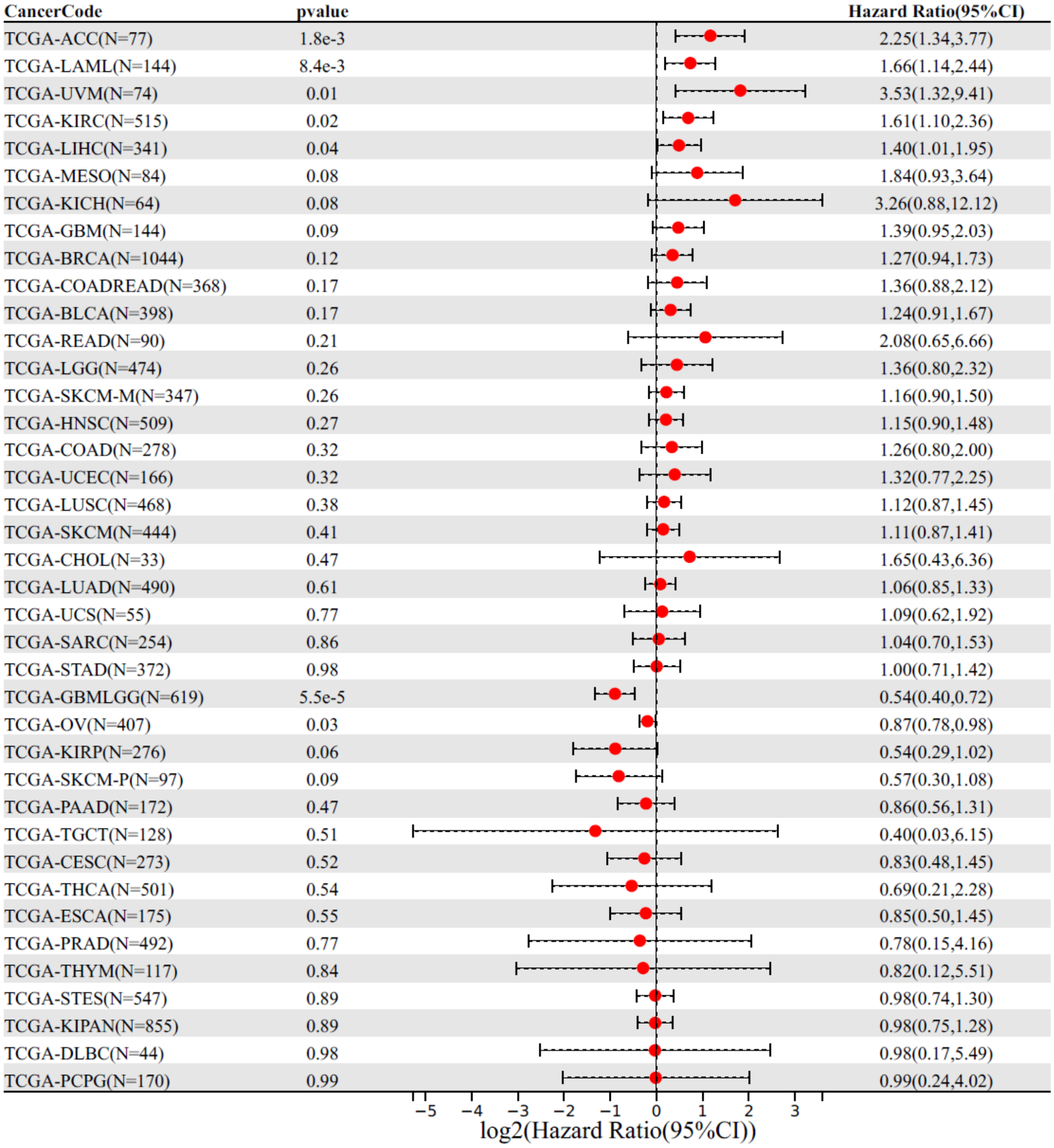


Figure S2. The HR values of SCYL1 in pan-cancer.

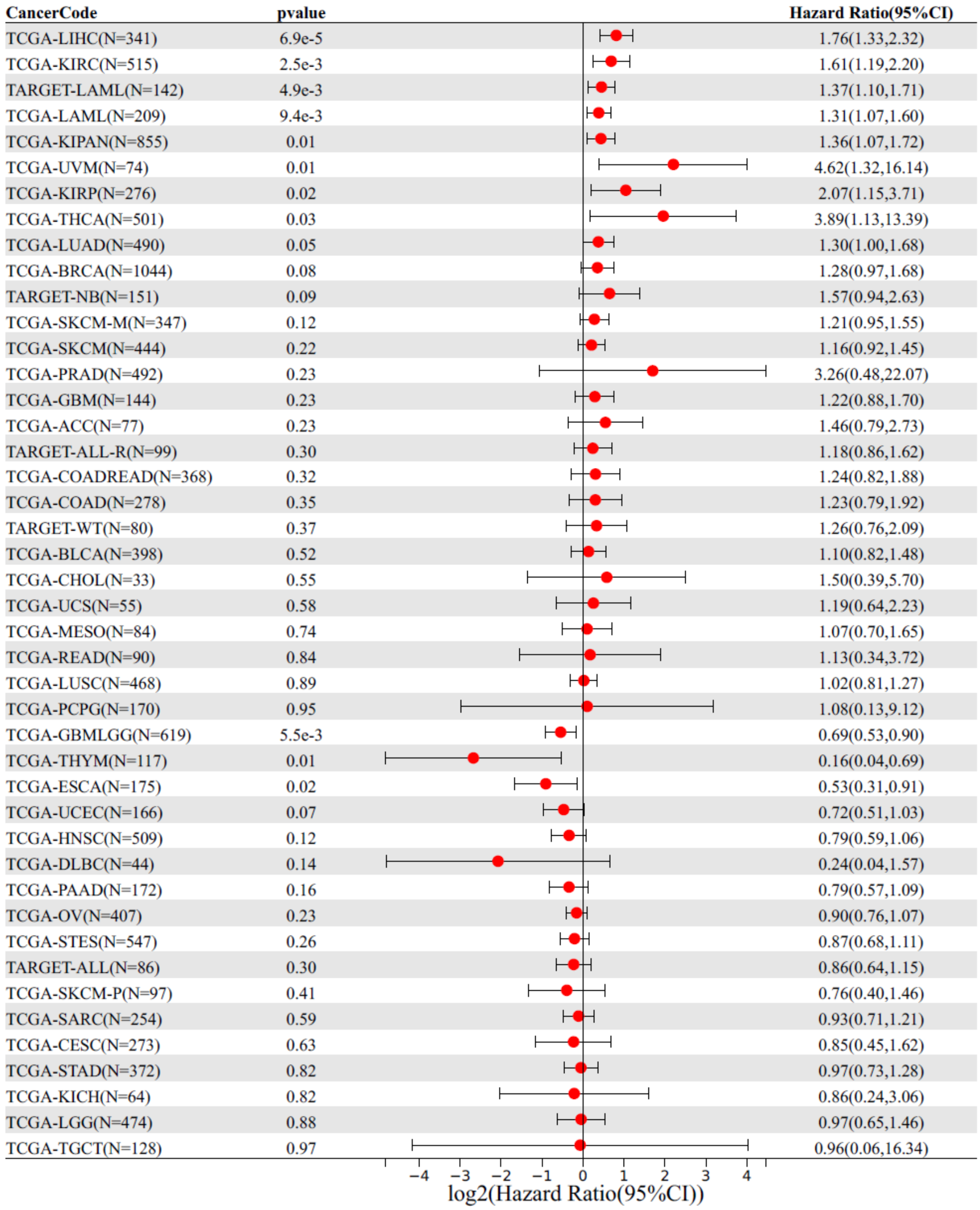


Figure S3. The HR values of ZNF668 in pan-cancer.

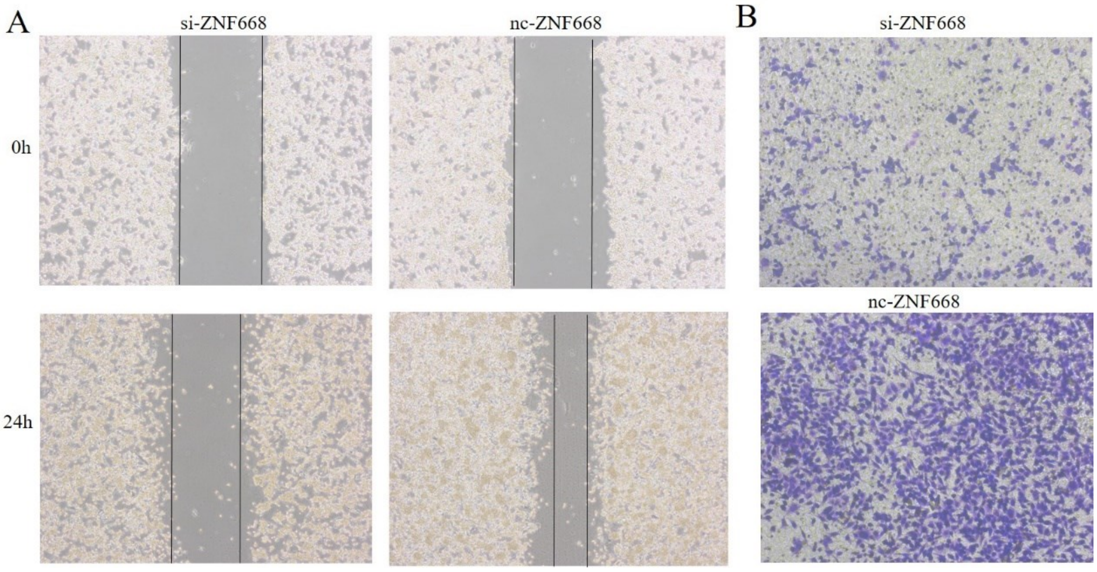


Figure S4. The ZNF668 oncogene role in leukemia. Would healing assays (A) and Transwell assays (B) results for ZNF668 in K562 cells.

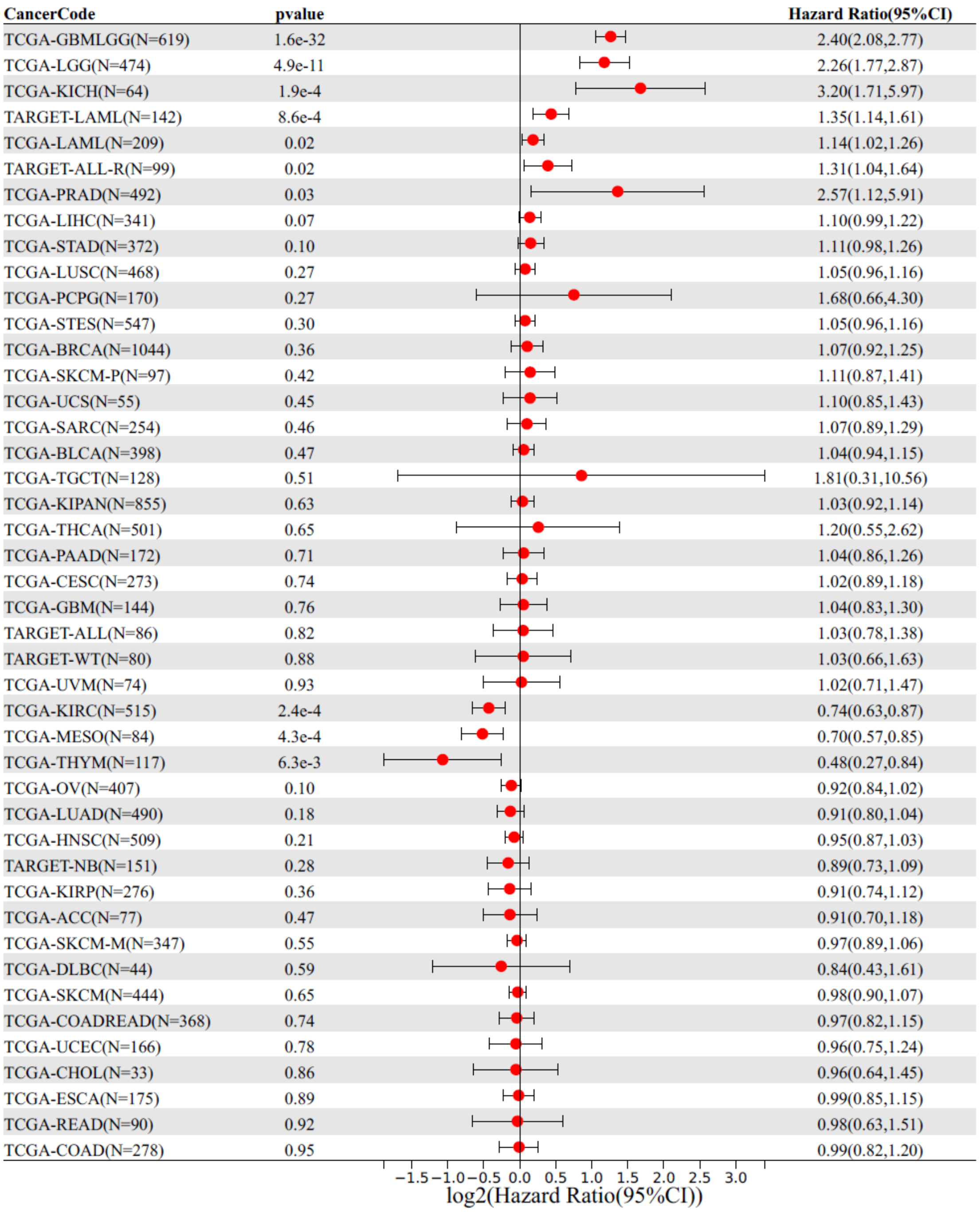


Figure S5. The HR values of ZNF788 in pan-cancer.

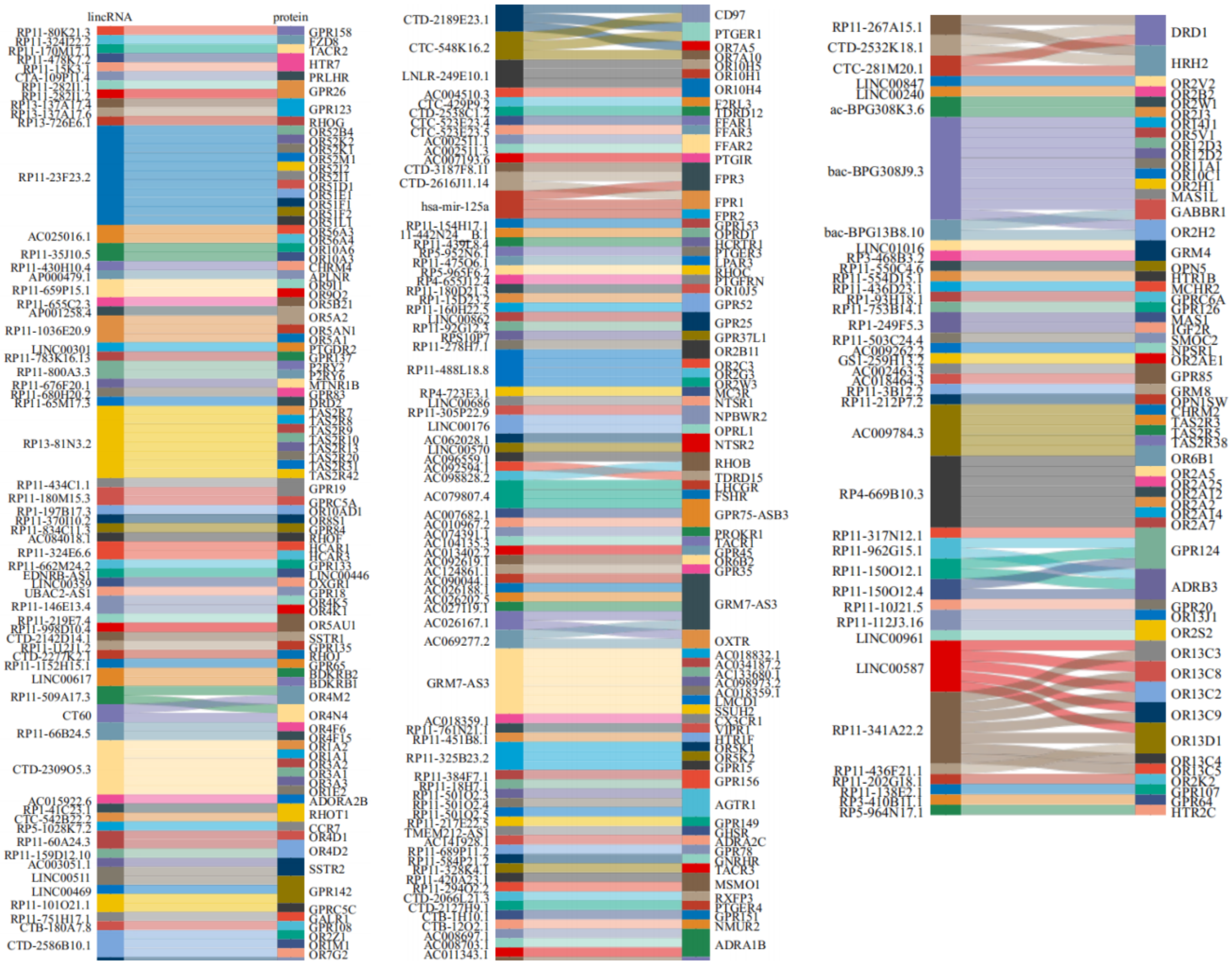


Figure S6. The details for the link between lincRNA and GPCRs in K562.

Table S1. Topological parameters for proteins with top 10 degree in the lincRNA-promoter network.

| Gene Name | Average shortest path length | Clustering | Closeness centrality | Degree | Betweenness centrality | Neibourhood connectivity | Express |
|---|---|---|---|---|---|---|---|
| CFB | 2.96 | 0.75 | 0.34 | 98 | 1.23E-04 | 51.47 | 0.24 |
| LTB4R2 | 3.06 | 0.73 | 0.33 | 78 | 8.74E-04 | 32.87 | 1.06 |
| DDX39B | 2.57 | 0.53 | 0.39 | 72 | 0.003143 | 51.87 | 176.50 |
| NELFE | 2.94 | 0.57 | 0.34 | 69 | 6.30E-04 | 47.24 | 31.16 |
| ZNF668 | 8.36 | 0.66 | 0.12 | 69 | 9.84E-06 | 37.63 | 1.55E-23 |
| NEU1 | 2.56 | 0.60 | 0.39 | 68 | 0.004752 | 53.58 | 15.61 |
| ZNF788 | 3.82 | 0.76 | 0.26 | 66 | 3.52E-04 | 34.38 | 0.34 |
| DXO | 2.57 | 0.58 | 0.39 | 65 | 0.005542 | 53.38 | 10.95 |
| MICB | 2.95 | 0.55 | 0.34 | 65 | 2.81E-04 | 46.83 | 7.35 |
| TP53 | 4.63 | 0.51 | 0.22 | 65 | 0.01231 | 38.98 | 63.52 |

Table S2. The mRNA expression values for the top 10 GPCRs.

| Gene Symbol | Median Tumor | Median Normal | Log2FC | P value |
|---|---|---|---|---|
| GRM7 | 9.39 | 5.59 | -0.75 | 2.40E-48 |
| AVPR2 | 4.47 | 0.11 | 2.301 | 0.00103 |
| CXCR2 | 3.75 | 0.06 | 2.164 | 0.00574 |
| F2RL3 | 0.17 | 8.09 | -2.958 | 3.03E-20 |
| GPR132 | 10.37 | 0.575 | 2.852 | 0.00125 |
| OPRL1 | 5.14 | 0.43 | 2.102 | 5.56E-07 |
| P2RY11 | 16.13 | 3.755 | 1.849 | 0.00363 |
| PTAFR | 18.09 | 0.49 | 3.679 | 0.000484 |
| RXFP4 | 4.07 | 0.05 | 2.272 | 0.00000609 |
| VN1R1 | 2.56 | 0.11 | 1.681 | 0.0000426 |

Table S3. The topological parameters for the top 10 GPCRs.

| Gene name | Average shortest path length | Clustering | Closeness centrality | Degree | Betweenness centrality | Neibourhood connectivity | Express |
|---|---|---|---|---|---|---|---|
| GRM7 | 2.47 | 0.72 | 0.41 | 43 | 0.00068 | 112.09 | 0.09 |
| VN1R1 | 4.10 | 0.79 | 0.24 | 34 | 0.0000136 | 43.03 | 0.06 |
| AVPR2 | 2.76 | 0.48 | 0.36 | 22 | 0.00324021 | 28.05 | 0.27 |
| OPRL1 | 3.47 | 0.64 | 0.29 | 22 | 0.0000633 | 27.64 | 0.42 |
| RXFP4 | 7.62 | 0.77 | 0.13 | 22 | 0.0000012 | 24.59 | 0.10 |
| P2RY11 | 4.13 | 0.71 | 0.24 | 20 | 0.00190419 | 22.95 | 18.78 |
| GPR132 | 3.04 | 0.68 | 0.33 | 19 | 0.000022 | 24.68 | 14.94 |
| F2RL3 | 3.73 | 0.79 | 0.27 | 17 | 0.0000967 | 35.29 | 0.20 |
| CXCR2 | 2.99 | 0.77 | 0.33 | 13 | 0.00000496 | 48.77 | 0.05 |
| PTAFR | 8.99 | 0.76 | 0.11 | 12 | 0.00000046 | 16.58 | 5.04 |

Table S4. LincRNA regulates mRNA by competing the miRNA binding site with mRNA

| LincRNA | Interaction miRNA ID | Energy | Software Evidence |
|---|---|---|---|
| EGOT | hsa-miR-10400-5p | -46.61 | miRanda, TargetScan |
| | hsa-miR-762 | -46.09 | miRanda, TargetScan |
| | hsa-miR-6775-5p | -45.82 | miRanda, TargetScan |
| | hsa-miR-1343-5p | -45.75 | miRanda, TargetScan |
| | hsa-miR-4767 | -45.47 | miRanda, TargetScan |
| | hsa-miR-6089 | -45.26 | miRanda, TargetScan |
| | hsa-miR-6815-5p | -44.77 | miRanda, RNAhybrid |
| | hsa-miR-4707-5p | -44.09 | miRanda, TargetScan |
| | hsa-miR-6724-5p | -43.53 | miRanda, TargetScan |
